# Melioidosis in Mauritius

**DOI:** 10.3201/eid1101.040605

**Published:** 2005-01

**Authors:** Mohammad Iqbal Issack, Chundr Dutt Bundhun, Hemraj Gokhool

**Affiliations:** * Victoria Hospital, Candos, Mauritius

**Keywords:** Melioidosis, Burkholderia pseudomallei, Mauritius, Rain, Systemic Lupus Erythematosus, Meteorological Factors, Immunocompromised Host, Septicemia, dispatch

## Abstract

We report the first case of human melioidosis from Mauritius, where *Burkholderia pseudomallei* has never been isolated. The patient was immunocompromised, had never traveled abroad, and had a history of regular exposure to mud. She became ill at a time when rainfall was higher than the monthly average.

Melioidosis is an infectious disease of humans and animals caused by *Burkholderia pseudomallei* (*1*). Most documented cases occur in Southeast Asia and northern Australia (*2*). In recent years, melioidosis has increasingly been recognized in the Indian subcontinent (*3*). Only a few cases have been reported in South America (*4*) and Africa (*5*). In the islands of the southwest Indian Ocean, although no human cases have been reported, *B. pseudomallei* has been isolated from pigs in Madagascar as far back as 1936 (*6*) and from the soil in Madagascar and La Reunion (*7*). We report the first case of human melioidosis in the southwest Indian Ocean island of Mauritius, where *B. pseudomallei* has not been isolated previously.

## The Case

A 40-year-old patient was admitted to the hospital on January 29, 2004, with fever, generalized weakness, diarrhea, and vomiting. Her temperature on admission was 39.2°C. Results of physical examination were unremarkable. Initial blood tests showed hemoglobin level of 8.5 g/dL, leukocyte count of 4.9 x 10^9^/L, and platelet count of 110 x 10^9^/L. Erythrocyte sedimentation rate was elevated at 88 mm/h. Her serum glucose was 8.5 mmol/L, and urea and electrolytes were normal. She was started on intravenous ciprofloxacin, but her fever persisted, and she became increasingly confused. Two days after admission, therapy was changed to cefotaxime and metronidazole. A blood culture was collected the next day. The following day, she had cellulitis of the right leg. She remained feverish and intermittently drowsy and confused. She died 9 days after being admitted. Her old hospital records became available shortly after her death, and we noted that she had been diagnosed with systemic lupus erythematosus (SLE) in 1994. When she last attended the outpatient department 3 months before her hospital admission, she was prescribed 50 mg azathioprine and 5 mg prednisolone daily. She was a housewife and lived in Cité La Cure, a poor suburb of the capital city Port-Louis. She had never traveled abroad. According to her mother, her home becomes very muddy after heavy rainfall, and her feet were often in mud while performing her household duties.

After 5 days of incubation, an oxidase-positive, gram-negative bacillus was isolated from blood cultures. It produced colonies that appeared dry and rugose on the plates after 48 h and was identified as *B. pseudomallei* by using API 20NE (BioMérieux, Marcy l’Etoile, France) with the profile 1156577. Antimicrobial susceptibility testing by disc diffusion showed the organism to be resistant to colistin, ampicillin, cephalexin, gentamicin, and ciprofloxacin and susceptible to co-amoxiclav, tetracycline, cefotaxime, ceftriaxone, ceftazidime, piperacillin, and meropenem. A large zone of inhibition was seen around the co-trimoxazole disc, within which a thin film of growth was observed.

## Conclusions

This case represents the first time *B. pseudomallei* was isolated in Mauritius. The patient must have been infected in Mauritius because she never traveled abroad. We are not aware of any study looking for the organism in soil in this country. Veterinary cases do not appear to have been reported previously in Mauritius (V.B. Groodoyal, pers. comm.). Whether human cases of melioidosis have been missed in the past is not known, and cases may be missed currently. Recognizing the disease depends on awareness on the part of clinicians and on the ability of microbiology laboratories to identify the causative organism (*1*,*8*). Before 1998, oxidase-positive, gram-negative bacilli other than *P. aeruginosa* were not identified to species level in laboratories in Mauritius. Since then, at our laboratory, which receives specimens for bacteriologic investigations from all government healthcare institutions, such organisms are routinely identified by API 20NE when isolated in pure culture from blood, but only occasionally when isolated from nonsterile sites such as sputum and pus swabs. Thus, nonsepticemic cases of melioidosis in Mauritius could easily have been missed. Diagnosis also depends on appropriate specimens being sent to the laboratory. Some clinicians routinely request blood cultures from patients with high fever before starting antimicrobial drugs, although in practice, the specimen is often collected by nursing staff after the first dose has already been administered. Other clinicians only request blood cultures if fever persists after a few days of empiric antimicrobial therapy. In the case reported here, prior administration of cefotaxime may have delayed *B. pseudomallei* culture from blood until 5 days of incubation, when the median time to obtain a positive blood culture result is typically 48 hours (*1*).

An association between rainfall and melioidosis has long been recognized; most cases in Thailand (*9*) and northern Australia (*10*) occur during the wet season. The increased number of cases noted during the rainy season may be caused by the movement of *B. pseudomallei* from deeper layers toward the surface when dry topsoil is moistened by rainfall (*2*).

In Mauritius, the rainy season is December to March. In January 2004, 196 mm rainfall was recorded in Port-Louis, which is 37% higher than the 1971–2000 mean rainfall for the region during this month. January 2004 was the sixth wettest January of the past 30 years in Port-Louis. Similarly above-average rainfall was recorded throughout the island in 2004.

Recent reviews have suggested a predominant role for percutaneous *B. pseudomallei* infection in the pathogenesis of melioidosis (*11*). Studies carried out in regions where melioidosis is endemic have shown that exposure to wet soil and water are associated with increased risk for disease (*9*). The feet of our patient were regularly exposed to wet soil during rainy periods.

In melioidosis-endemic areas, although a large percentage of the population has been exposed to *B. pseudomallei*, as determined by seroprevalence studies, only a few develop melioidosis (*12*). Most cases occur in patients with underlying illnesses, such as diabetes mellitus, renal disease, and alcoholism (*9*,*10*) or in those who are immunosuppressed (*1*). Our patient had SLE and was on immunosuppressive drugs. Septicemic melioidosis has been reported in patients who have SLE (*13*).

This first case of melioidosis in Mauritius occurred in an immunosuppressed patient who had a history of prolonged and regular exposure to mud during a year when rainfall was higher than average. This combination of 3 risk factors does not occur regularly, and it is possible that few additional cases will be recorded in Mauritius in the future. However, clinicians and laboratory staff must remain aware of this disease, particularly because in a noncommunicable disease survey carried out in 1998, almost 20% of the Mauritian population >20 years of age were found to have type 2 diabetes mellitus (*14*), the most common predisposing condition for melioidosis (*1*). Determining the distribution of *B. pseudomallei* in soil in Mauritius by conducting environmental investigations will also be useful.
